# An Impact of COVID-19 on Cancer Care: An Update

**DOI:** 10.3390/vaccines10122072

**Published:** 2022-12-04

**Authors:** Vivek P. Chavda, Feng-Feng Ping, Zhe-Sheng Chen

**Affiliations:** 1 Department of Pharmaceutics and Pharmaceutical Technology, L. M. College of Pharmacy, Ahmedabad 380009, India; 2 Department of Reproductive Medicine, Wuxi People’s Hospital Affiliated to Nanjing Medical University, Wuxi 214023, China; 3 Department of Pharmaceutical Sciences, College of Pharmacy and Health Sciences, St. John’s University, Queens, New York, NY 11439, USA

**Keywords:** COVID-19, SARS-CoV-2, cancer, vaccine, monoclonal antibodies, immunocompromised patients

## Abstract

The world has been affected socioeconomically for the last two years due to the emergence of different variants of the COVID-19 virus. Vaccination is the major and most efficient way to prevent the widening of this pandemic. Those who are having comorbidities are more vulnerable to serious infections due to their immunocompromised state. Additionally, cancer patients could be at significant risk for COVID-19. In this pandemic era, the diagnosis and treatment of cancer were significantly affected. Clinical trials at the initial stage were performed on healthy or COVID-19 infected patients. This produces a greater level of hesitancy in cancer patients. This review article provide an update regarding the vaccination and treatment for COVID-19 in patients with cancer and future directions.

## 1. Introduction

**Figure 1:**
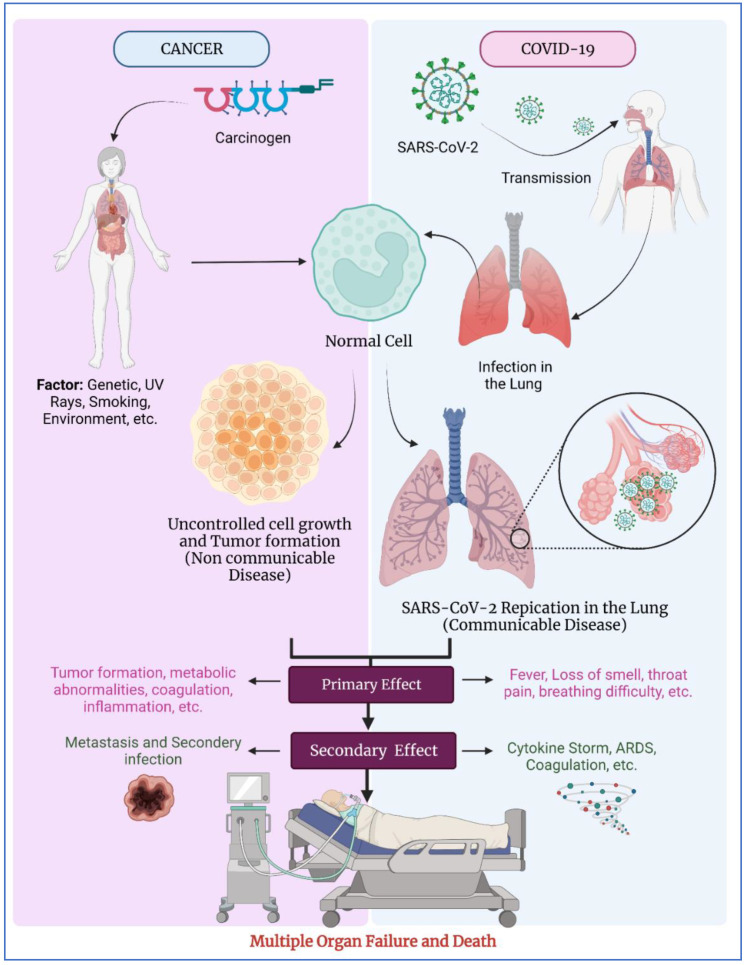
Connection between cancer and COVID-19. (Modified from [16] under CC BY 4.0 License).

The COVID-19 outbreak has caused catastrophe in health care systems, raising worries about its influence on non-COVID illnesses [1,2]. Cancer diagnosis and treatment are time-sensitive, and any delay is likely to have serious consequences for patients. Since no preparation strategy can completely defend against black-swan occurrences like the severe acute respiratory syndrome coronavirus 2 (SARS-CoV-2) outbreak, it is crucial to guarantee that such incidents do not lead to the collapse of critical healthcare sectors. During the COVID-19 pandemic, the World Health Organization (WHO) conducted a Global Pulse Survey that looked at the continuity of essential health services. According to the report, between 5 and 50% of cancer care-screening and treatment was disrupted in all countries as reported in the last quarter of 2021 [3]. This is despite the improvement since the first quarter of last year, in which service disruptions exceeded 50% in 44% of countries, and ranged between 5 and 50% in the rest [3]. The complex and delayed reaction to COVID-19 serves as a wake-up call to governments throughout the world that healthcare must be emphasized worldwide, and that affordable healthcare might be a fundamental right that cannot be neglected [4]. Almost 3–4 percent of all COVID-19 victims have cancer and related diseases, and the numbers are terrifying, as the patient is described as “doubly unlucky” to be suffering from cancer as well as being infected with COVID-19. Cancer patients could be at significant risk for COVID-19, and there seems to be an upsurge in the likelihood of serious conditions, such as ICU usage and artificial ventilation, for them as opposed to non-cancer COVID-19 patients [5]. Other than cancer, people who are suffering from medical conditions such as cardiovascular disease, diabetes, and, chronic respiratory disease are also more likely to develop serious illnesses [6]. The immunocompromised status of patients is almost certainly the reason for this elevated danger to cancer victims with COVID-19. The lethal combination of COVID-19 and cancer is also being blamed on cytokine storms. Patients with cancer and COVID-19 both have inflammatory responses [7]. Because the inflammation is pervasive, the misery of cancer patients is exacerbated. Additionally, many molecular and biological processes are also reported in patients with cancer, such as a cytokine storm, increased angiotensin-converting enzyme 2 (ACE2) and transmembrane protease serine 2 (TMPRSS2) production, in addition to coagulopathy [8], which is a potential concern observed in a lot of cancer patients (Figure 1). In respiratory system cells, the increased amount of Anexelekto (AXL) is also reported. It plays an important role in cancers and in COVID-19 as a transmembrane protein that promotes cell growth, migration, aggregation, metastasis, and adhesion [9]. The management and cure of cancer is handled by many nanotechnology based products. [10,11,12]. Herbal drugs are converted to nano pharmaceuticals to provide effective cancer management [13,14,15].

This review aims to highlight the requirement for substantial clinical proof of the efficacy and safety profile of several COVID-19 vaccines in order to motivate patients to get immunized. To give the best care, the impact of SARS-CoV-2 on the different forms of cancer differs and has to be thoroughly researched. The review makes the claim that it will help readers comprehend how cancer and other co-occurring disorders are linked to COVID. It also focuses on the hesitancy, its cause, and the necessary precautions to be undertaken before receiving a vaccine.

## 2. Cancer Diagnostics and COVID-19

According to Allegra et al. [17], COVID-19 and carcinoma have similar markers such as the carbohydrate antigens (CA) 125 and 153, carcinoembryonic antigen (CEA), human epididymis protein 4 (HE4), C-reactive protein (CRP), and cytokeratin-19 fragment (CYFRA21-1); However, establishing whether the increase in such biomarkers is the result of COVID-19, malignancy, or both at the same time is a challenge for hospitals and clinicians (Figure 1) [14]. The importance of diagnosis cannot be overstated, and it necessitates broad information on the pathophysiology of the lethal pair of COVID-19 and malignancy. According to one study, the broad impact of COVID-19 on cancer patients and the system that assists them may cause mortality among cancer patients in the United Kingdom to increase by 20% over the course of the following 12 months, resulting in 6000 more deaths. [18]. As per Englum et al. [19], There are 4.1 million cancer-related interactions, 3.9 million applicable operations, and 251,647 new malignancies diagnosed between 2018 and 2020. In 2020, colonoscopies declined by 45 percent compared to the yearly averages from 2018 to 2019, while prostate biopsies, chest computed tomography scans, and cystoscopies decreased by 29%, 10%, and 21 %, respectively [13]. The number of new cancer diagnoses fell from 13% to 23%. Despite decreases in pandemic-related limitations, these declines varied by region and continued to accrue. Therefore, “Post-pandemic cancer diagnoses across races/ethnicities should be tracked to see whether measures to boost equality in cancer detection and early treatment are bearing fruit”, noted Caswell-Jin [20]. In one another study, a total of 10,404 participants in the cohort of 23,266 cancer patients and 1,784,293 cancer-free participants reported shows results like a positive COVID-19 test. Cancer survivors had a 60% increased risk of a positive COVID-19 test compared to those without cancer. The odds of a positive test were 2.2-fold higher among patients with cancer receiving chemotherapy or immunotherapy. Among participants over 65 years of age and males, COVID-19 infection was associated with a higher risk of cancer [21]. In the United States, cancer-related mortality rose by 3% during the pandemic era, most likely as a result of delayed diagnoses and inefficient patient care that forces hospitals to treat patients at high risk of contracting COVID-19. According to the retrospective research of two institutes, a positive COVID-19 test caused a delay in the length of cancer therapy. The length of the delays varies depending on a number of factors, including time between test results, a string of positive tests, fear of medical treatment and hospitalization, etc. A total of 131 cancer patients who were infected with COVID required hospitalization, of whom 38% required ICU care, and 7% died [22].

## 3. COVID-19 and Cancer Treatment

Cancer patients are more likely to be hospitalized and die after contracting SARS-CoV-2 [23]. As a result, we sought to undertake one of the first population-level analyses of vaccination efficacy against SARS-CoV-2 outbreaks in cancer patients [24]. Chemotherapy and immune suppressants used after surgical cancer excision typically damage the patient’s immune system. This implies that COVID-19 may harm any such patient more than it would a healthy person. Children who get anti-cancer medication and hematopoietic stem cell transplantation (HSCT) have an increased risk of serious complications (up to 20%) and death (4–5%). The majority of these data come from the registry or institution-based sources and predate the Delta/Omicron variants’ dominance [25]. The therapy must be carried out while keeping the extra COVID-19 risk factors connected with cancer in mind. Certain anti-cancer therapies may necessitate a reduction in immunity to destroy cancer cells that provide a fair chance of SARS-CoV-2 infecting such patients. To prevent patients from experiencing the lethal combination of COVID-19 and cancer further, physicians and cancer organizations recommend delaying cytotoxic treatment until COVID negative diagnostic results in such infected patients [26,27,28].

Human tissues other than the lungs express TMPRSS2 and ACE2, including the testes, which are susceptible to SARS-CoV-2 infection. A very common alteration in prostate cancer is the fusion of TMPRSS2 with another androgen-responsive gene. In their study, Bhowmick NA et al. emphasized the evidence that TMPRSS2 and ACE2 are affected by androgens, as well as the implications of using androgen suppression to inhibit TMPRSS2 to target SARS-CoV-2 [29].

Cancer treatment is primarily provided by pre-pandemic standards in regions with a low prevalence of viral infection. It is often advised that treatment be postponed until it is certain the patient will not get COVID-19 when a patient is known to have been infected with SARS-CoV-2, especially if he or she has not had a COVID-19 vaccination or if the vaccine is anticipated to have an unsatisfactory response. Patients undergoing low-risk cancer medicines, such as hormone medications, may be exempt from receiving therapy [30]. For patients with low-viral transmission systemic therapy, radiation therapy and surgery have been undertaken [31].

Cancer treatment in locations with strong viral transmission-the parts that follow will focus on the challenges associated with systemic anticancer medicines, radiation therapy, and surgery in regions with high rates of persistent viral transmission during the pandemic’s peak times of activity. In areas where the virus is less prevalent, all of these characteristics are less significant [32].

Barrière et al. provide five suggestions to health authorities and healthcare professionals [33].

Statement 1: All cancer patients should have a third vaccination dose three to four months after receiving the second dose (Dose 2), along with controls for any remaining anti-S serum antibodies, as cancer patients have a lower median anti-S antibody titer after two vaccine doses than the general population. The data currently available demonstrate quicker Omicron VOC immunity fading than the Delta strain, but enhanced protection following booster dosing (Dose 3) [34].

Statement 2: Decide that if a patient’s serum anti-SARS-CoV-2 S (antiS) titer falls below 1000 BAU/mL, the fourth dose of vaccination should be suggested. This cut-off corresponds to titers seen in a group of individuals without comorbidities 3–4 months after Dose 2, for whom Dose 3 treatment is currently recommended in the majority of industrialized countries to give the best protection against the Omicron variant.

Statement 3: Monoclonal antibody (mAB) tixagevimab/cilgavimab (EVUSHELD, AstraZeneca) is used for pre-exposure prophylaxis for cancer patients who do not have seroconversion after Dose 3 or 4. This combination of long-acting anti-S mAbs has a remarkable effect on in vitro efficacy of Delta VOC and is less affected by several Omicron S mutations [33,35].

Statement 4-A three-dose complete vaccination for the patient’s family should be suggested as soon as 4 months following Dose 2. Additionally, when in close, frequent contact with immune-compromised parents, the majority of Scientific Pediatric Societies, as well as the US (Food and Drug Administration) and European Medicines Agency (EMA) Health Agency advise the immunizing children between the ages of 5 and 11 [36].

Statement 5: Patients should use a high-protection mask that filters at least 95% of particles(N95) and has a filtering facepiece type 2 (FFP2). Such masks should be required in crowded environments, whether on public or private transport (when shared with others). Recent comparative data demonstrates that these masks offer more effective personal protection than surgical masks [37].

## 4. COVID-19 Vaccine and Cancer

According to current prime-boost theories, patients with cancer can receive the COVID-19 vaccine in a safe and therapeutically effective way [38,39,40]. The COVID-19 vaccines are considered efficacious against the majority of viral variants, such as the Delta form. Variants may, nevertheless, promote disease in certain vaccinated persons. The Omicron form, for example, is more pathogenic than the Delta variant and may produce recurrent infections in vaccinated patients. Booster dosages are critical in lowering the chance of a reoccurring illness [41,42,43]. Slomski reported [44] that the trial, which was undertaken with the Pfizer COVID-19 vaccine in Israel, included 232 cancer patients along with a control group of 261 healthcare workers. The results after the administration of the first immunization are average, but after the second vaccine, almost 86% of the cancer patients demonstrated sufficient neutralizing antibodies against SARS-CoV-2. Experts agree that COVID-19 immunization is appropriate for cancer patients, survivors, and those receiving cancer treatment, including chemoimmunotherapy [45]. The best current information shows that if you have cancer, your chances of dying or having serious problems with COVID-19 are approximately two times higher than those with no cancer. Until now, patients receiving cancer therapies had been precluded from COVID-19 vaccination studies. Our presumptions are thus based on what we understand about the safety and effectiveness of the finest vaccine candidates, the assessment of conventional vaccination in patients with cancer, and the immunological alterations brought on by current cancer treatments. To increase the effectiveness of the vaccine in immunocompromised cancer patients, nanotechnology must be used as a means of building a successful vaccine platform that can be manufactured in a scalable manner and distributed worldwide as well as delivered faster and more precisely [46]. The study team found that 79% of vaccine-treated cancer patients displayed a T-cell response, and comparable findings were observed in patients with solid tumors and blood cancers. It is noteworthy that even with the delta version, the vaccines were seen to activate T cells in those who had not developed a neutralizing antibody response by the time they received the second dose [47]. According to the findings of Obeid et al. [48], with a comparative efficacy study, there are neutralizing antibodies (nAbs) after two doses of vaccine in 50% of a cancer patients that seems to lose the protection after six months of immunization. These findings might aid in the development of the optimum boosting immunization regimen, particularly for immunocompromised individuals. Fendler et al. [49] conducted CAPTURE research and demonstrated that “Previous SARS-CoV-2 infection boosted the nAb response including against VOC, and anti-CD20 treatment was associated with undetectable nAbT. Vaccine-induced T cell responses were detected in 80% of patients and were comparable between vaccines or cancer types”.

New observational research published by Sheeba Irshad et al. involved 151 cancer patients, including 95 with organ cancer and 56 patients with hematological cancer [50]. There were no documented vaccine-related fatalities. One administration of the BNT162b2 vaccination is ineffective in cancer patients. Immunogenicity was dramatically enhanced in patients with solid tumors after 2 weeks of a vaccination boost on day 21 following the first dose. These findings support the prioritizing of cancer patients for an early (day 21) second BNT162b2 vaccination. According to a new study, the Omicron variant of SARS-CoV-2 is more hazardous in cancer patients than in the general population. Despite this, the death rate among cancer patients infected with omicron is lower than in prior SARS-CoV-2 variants [51]. More research focusing on strategies to boost vaccination efficacy and the identification of novel treatment options is critically required for these patients. Three months following the second immunization dose (Dose 2), all cancer patients should get a third dose of the vaccine, along with any further serum anti-S antibody controls [36].

## 5. Vaccines and Their Ongoing Clinical Trials

An international panel of oncology experts examined the long-term consequences of the pandemic on cancer clinical trials using new data on worldwide clinical trials gathered by IQVIA, and suggests remedies for clinical trial stakeholders [52]. Oncology trials may be time-consuming, costly, and stressful for patients and their families, complicating the decision to enroll even before concerns about COVID-19 arose [53].

Patients who have had malignant tumors may have diminished immunity as a result of immunosenescence, previous treatment, or comorbidities, which will affect the effectiveness of COVID-19 vaccines [54]. People with active cancer and people taking immunosuppressive medicines, however, were excluded from some vaccination trials, which questions their effectiveness and safety [18]. A significant portion of participants in the randomized, placebo-controlled, observer-blinded phase 1/2/3 BNT162b2 mRNA COVID-19 vaccination trial had a history of neoplasm at baseline, either past or present [55]. This study will also offer a post hoc subgroup analysis of clinical efficacy and safety in persons with a history of neoplasm from this experiment in response to the need for solid confirmation of vaccination effectiveness and toxicity in cancer patients [56]. Our post hoc subgroup analysis of the clinical efficacy and safety of this vaccine in individuals who have a history of malignancy fulfills the requirement for direct proof of vaccination efficacy and safety in cancer patients [57]. BNT162b2 was 95% effective in preventing COVID-19 in people 16 years of age from 7 days post-dose two after a median follow-up of 2 months in the overall study group. Later studies in teenagers (aged 12 to 15) revealed 100% efficacy and non-inferior immunogenicity compared to young adults (aged 16 to 25) [58]. For dose two, the updated vaccination efficacy throughout the whole trial cohort was still high (91%) after up to 6 months of follow-up. Based on these findings, the US FDA granted emergency use authorization for the BNT162b2 COVID-19 vaccine in December 2020, and the European Union granted conditional marketing clearance for the immunization of individuals 12 years of age and older [59]. Recently, mRNA-1273 or BNT162b2 mRNA vaccines have been reported to increase resistance to neutralizing antibodies of the SARS-CoV-2 Omicron variant. However, an additional, third dose of the mRNA booster vaccine appears to provide much stronger protection against Omicron, similar to what has been reported for other variants of concern [60]. A patient with cancer who develops breakthrough COVID-19 following a full vaccination remains susceptible to severe outcomes [61]. On 23 August 2021, the FDA approved the BNT162b2 COVID-19 vaccine after receiving a biologics license application for individuals 16 years of age and older. Trial participants who were not using immunosuppressive drugs at baseline but had a prior prognosis of any cancer or other neoplasm were included in the current post hoc subgroup analysis [62]. Being almost neglected during the initial trials of the COVID-19 vaccine, healthcare is healthcare is now focusing on the immunosuppressed patients as well. Cancer patients who have received two doses of the mRNA vaccine (Spikevax and Comirnaty) are the subjects of recruitment research. The focus of the study is on how cancer patients’ humoral immunity develops (NCT05075538). Patients with solid tumors, multiple myeloma, and hematologic malignancy were included in a phase-2 study of mRNA-1273, which demonstrated the drug’s immunogenicity and safety profile (NCT04847050). On the patient with malignant solid neoplasm, another observational study is carried out to determine the safety and effectiveness of the vaccination. Its major consequence, antibody production, and t-cell-mediated immunization are the outcome criteria. The investigation should be finished by 31 December 2023. (NCT04865133). The long-term safety of patients who will be subjected to therapy that might lower their immunity is a serious worry. As COVID-19 targets lung cancer, the situation there might deteriorate. An observational trial of 300 lung cancer patients focused on the long-term impact and safety profile of the vaccination (NCT04894682). Immunotherapy is one of the major approaches for the treatment of cancer. A study on 53 patients was performed to evaluate the immune response produced in cancer patients exposed to immunotherapy and normal cancer patients (NCT05062525).

Vaccine hesitancy is more prevalent in malignant patients as compared to the normal individual. A study conducted on 429 patients concluded that 48% of the population was unsure regarding the vaccine consumption primarily due to safety concerns (32%) and about the complication it might cause (12%) [63]. According to a different study on leukemia patients and survivors, 17% of all responders were unlikely to receive the vaccination. The dread of their health condition getting worse and worries about the safety of the vaccination were two potential causes [46,64].

## 6. Discussion

A cancer patient receives many treatments, the bulk of which target the immune system. According to a research, cancer patients have a greater fatality rate and need for intensive care than healthy people do. Immunosuppression in patients limits the use of live vaccines due to an increased risk of infection. However, immunization is necessary to guard against novel variations. If the patient is commencing chemotherapy, they should allow at least 14 days before the immunization. A patient with blood cancer who is receiving chemotherapy should not be vaccinated until the neutrophils achieve homeostasis. This circumstance has caused cancer therapy to be postponed or interrupted. This element may make the malignancy worse and, in extreme situations, may be lethal. According to clinical research on 18 cancer patients who had recently contracted COVID-19, the fatality rate for cancer patients had increased to 5.6% from 2.3%. This emphasizes that in order to offer proper care and maintain patient safety, a thorough investigation regarding the effects of real illness and vaccination must be conducted.

## 7. Concluding Remarks

The COVID-19 pandemic encourages us to develop viable strategies for dealing with subsequent health crises [65,66,67,68,69,70]. However, there was some data redundancy or duplication in tests and experiments due to the heterogeneity in inclusion criteria between distinct cancer registries that were restricted to different subgroups or regions. Going forward, coordinated standardized initiatives in which various registries collaborate to build a uniform method for consistent data collecting and reporting could yield speedier, more precise, and more robust clinical implementation outcomes. More research is required to prove the safety and efficacy of therapeutic molecules in COVID-19 with comorbidities such as cancer. Future medical science demands a better understanding of various drug-drug and drug-pathological interaction and develops preventive measures to ensure the wellbeing of the patient.

## Data Availability

Not applicable.
